# Detection of Androgen Receptors in Spermatozoa of Small Ruminants: A Putative Modulation Pathway for Cryoresistance Through AQP3

**DOI:** 10.3390/ijms252211972

**Published:** 2024-11-07

**Authors:** Esther Alba, Cristina Castaño, Adolfo Toledano-Díaz, Rosario Velázquez, Belén Martínez-Madrid, Alberto Gómez-Crespo, Manuel Álvarez-Rodríguez, Heriberto Rodriguez-Martinez, Julián Santiago-Moreno

**Affiliations:** 1 Department of Animal Reproduction, Spanish National Institute for Agricultural and Food Research and Technology (INIA-CSIC), 28040 Madrid, Spain; albasanchezesther@gmail.com (E.A.); cristina.castano@inia.es (C.C.); toledano@inia.csic.es (A.T.-D.); albego32@ucm.es (A.G.-C.); manuel.alvarez-rodriguez@inia.csic.es (M.Á.-R.); 2 Department of Animal Medicine & Surgery, Faculty of Veterinary Medicine, Universidad Complutense de Madrid (UCM), 28040 Madrid, Spain; belmart@ucm.es; 3 Department of Biomedical & Clinical Sciences (BKV), Obstetrics & Gynecology, Linköping University, SE-58185 Linkoping, Sweden; heriberto.rodriguez-martinez@liu.se

**Keywords:** aquaglyceroporins, androgen receptor, sperm, testosterone, ruminant

## Abstract

This work was aimed to identify androgen receptors (AR) in the spermatozoa of wild and domestic ruminants and to assess the effect of testosterone on sperm localization of aquaporin-3 (AQP3) and cryopreservation process. Sperm samples from wild species were incubated with testosterone (T group), 1,3-propanediol (PDO group), phloretin (PHL group), PDO+T group, PHL+T group. Western blot identified the presence of AR as a single band of about 48 KDa. Immunolabelling of AR was located in the equatorial segment of the sperm head. In mouflons, the cryoresistance ratio for acrosome integrity was lower (*p* < 0.05) in the PHL+T than in Control and T groups. In ibexes, the cryoresistance ratio for acrosome integrity was lower (*p* < 0.05) in the PHL+T, PHL, and T group than in the Control group; the cryoresistance ratios for sperm kinematic variables were lower (*p* < 0.05) in PDO+T than in Control. No changes were found among treatments in the proportion of spermatozoa showing AQP3 in the different membrane domains after incubation and thawing in both mouflon and ibex. In conclusion, testosterone negatively affected sperm cryoresistance expressed as acrosome integrity, enhancing the effects of the AQP blocker PHL. Our findings provide a sound knowledge of the molecular mechanisms that explain the seasonal variation in sperm freezability from ruminants.

## 1. Introduction

Androgens, i.e., testosterone and dihydrotestosterone (DHT), are steroid hormones essential for reproductive development and the establishment of male sexual characteristics, among other functions. In the testes, testosterone is the principal hormone regulating spermatogenesis. Produced by Leydig cells in response to luteinizing hormone (LH) stimulation, testosterone diffuses through the seminiferous tubules [1]. Testosterone is crucial for maintaining spermatogonia numbers, progression through meiosis, blood-testis barrier (BTB) integrity, seminiferous lumen formation, spermatid adhesion, and spermiation [2]. The enzyme 5α-reductase in target organs as the epididymis converts testosterone into the biologically more active DHT [3]. Testosterone and DHT mediate their actions through androgen receptors (AR). In contrast to other receptors (as for polypeptide hormones) [4] the AR is mainly located in the cytoplasm of a diverse range of cells [3]. In the absence of ligand, the AR is cytoplasmic; when androgens bind to the AR, the androgen/AR complex translocates to the nucleus to modulate gene transcription [3]. In addition, other studies reported that AR is also present in the cell membrane, where they can initiate intracellular signaling cascades [5], findings that initiated a debate regarding the presence and function of AR in spermatozoa. Functional AR expression has been identified in human, bank vole and pig spermatozoa [6,7,8,9], and the presence of testosterone binding sites on the sperm surfaces, which would determine a non-genomic effect, have been associated with motility in bonnet monkeys [10]. However, the presence and functionality of AR in ruminant spermatozoa remain unproven.

The secretion of testosterone in most male ruminant species fluctuates throughout the year due to factors such as photoperiod, which helps maintain optimal reproductive activity during the brief rutting season [11]. A negative correlation has been observed between high circulating testosterone levels and sperm freezability in sheep [12] and goat [13]. These findings are more marked in their respective wild ancestors, the Iberian ibex [14] and mouflon [12] but the underlying molecular mechanisms remain unclear.

Aquaporins (AQPs) are membrane proteins that facilitate water exchange between the cell interior and exterior [15]. AQP3, an aquaglyceroporin that allows the passage of water and solutes such as glycerol, plays a critical role in sperm osmoadaptation [16] and cryoresistance [17]. In rams, AQP3 is involved in changes in energy metabolism during sperm capacitation, which is affected by individual testosterone variations [18]. Testosterone exposure increases the expression and alters the distribution of AQP1, AQP5, and AQP7 in the uterine cells of female mice [19]. Additionally, testosterone regulates AQP4 in cultured astrocytes [20] and AQP9 in human prostate [21]. A possible role of androgens in the expression of AQP3 and AQP10 in different plasma membrane domains, that ultimately might affect cryoresistance [22], has been suggested.

Given these findings, the aim of the current study was to identify the presence and localization of AR in small ruminant spermatozoa, including those of wild ancestors of sheep and goats due to their marked rutting season with strong seasonal changes in plasma testosterone concentrations. Moreover, we ought to investigate the potential effect of testosterone on the localization of AQP3 in spermatozoa and its implications for the cryopreservation process in wild ruminant species.

## 2. Results

### 2.1. Identification and Localization of AR

**Figure 1:**
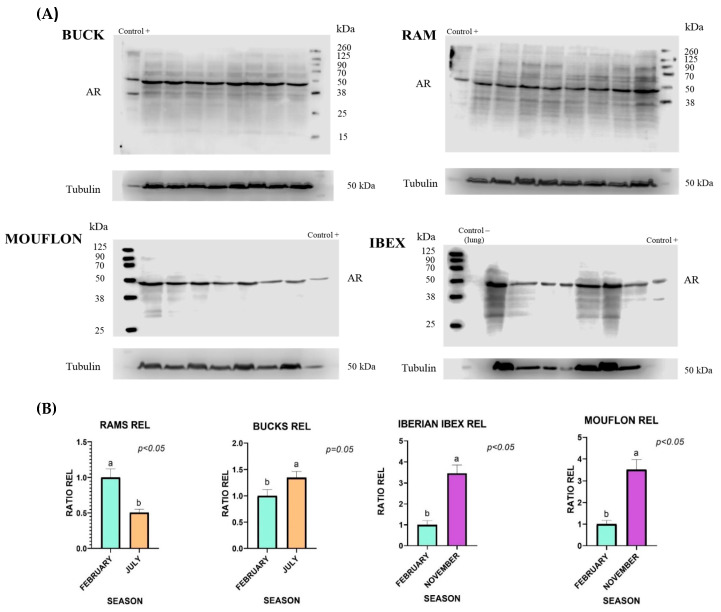
Identification of AR by WB. (**A**) AR was identified as a band of about 48 kDa in buck, ram, mouflon and ibex spermatozoa, coinciding with mouse vas deferens tissue lysate (Control+). (**B**) Relative abundance of AR bands (mean ± SEM) from samples collected at the onset (July and November) and the end (February) of the rutting. Different letters (a,b) indicate significant differences (*p* ≤ 0.05) between the onset and end of rutting after quantification of 48 kDa bands and normalization using tubulin protein as an internal standard.

**Figure 2:**
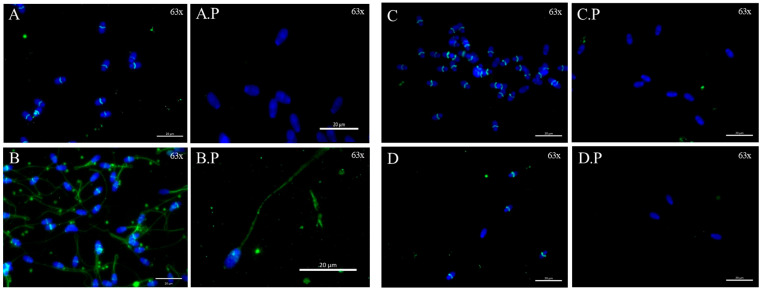
Immunolocalization of AR in small ruminant spermatozoa. Immunolabelling of AR in (**A**) ram, (**B**) buck, (**C**) mouflon, and (**D**) ibex in fresh spermatozoa. Sperm nuclei were stained with Hoechst (blue). Immunolabelling for AR (green) is located in the equatorial segment of the sperm head. Blocking peptide of AR in (**A.P**) ram, (**B.P**) buck, (**C.P**) mouflon, and (**D.P**) ibex. (**B.P**) Unspecific AR staining in the sperm tail.

WB identified the presence of AR as a single band of about 48 KDa in buck, ram, and their respective wild ancestors (ibex and mouflon). Relative abundance of AR bands varied according to the reproductive period. The abundance of AR was greater (*p* ≤ 0.05) at the onset of the rutting season in all studied species except for Merino ram, in which greater expression was found at the end of the rutting season (Figure 1). The location of the AR, mapped by ICF, showed that AR was located exclusively in the equatorial segment of the sperm head in buck, ram, mouflon and ibex (Figure 2). Immunofluorescence of the peptide competition assay for the anti-AR revealed unspecific AR staining in the sperm tail (Figure 2).

### 2.2. In Vitro Assay of Testosterone Effect on Cryoresistance and Expression of AQP3

After incubation of sperm samples with different treatments, based on testosterone and AQP3 inhibitors (control, testosterone (T), 1,3-propanediol (PDO), phloretin (PHL), PDO+T, PHL+T), the analysis of sperm variables revealed that PHL+T group showed the least favorable outcomes in mouflons. Specifically, the kinematic sperm variables (VSL, VAP, LIN, STR, WOB, and BCF) were lower (*p* < 0.05) in the PHL+T group than in the Control (Table 1).

In ibexes, after incubation, the least favorable kinematics variables were found in both PHL and PHL+T groups, without differences among them. Specifically, VCL, VSL, VAP, ALH, and BCF values were lower (*p* < 0.05) in PHL and PHL+T than in the Control group. The remaining sperm variables did not show differences in comparison with Control (Table 2).

In mouflons, the cryoresistance ratio for acrosome integrity (NAR) was lower (*p* < 0.05) in the PHL+T than in Control and T groups; conversely, the LIN parameter yielded greater values in these groups than in controls (Table 3).

In ibexes, the cryoresistance ratio for acrosome integrity was lower (*p* < 0.05) in the PHL+T, PHL, and T groups than in the Control group (Table 4). The cryoresistance ratio for VCL, VSL, VAP, ALH and BCF, was lower (*p* < 0.05) in PDO+T than the Control. (Table 4).

**Figure 3:**
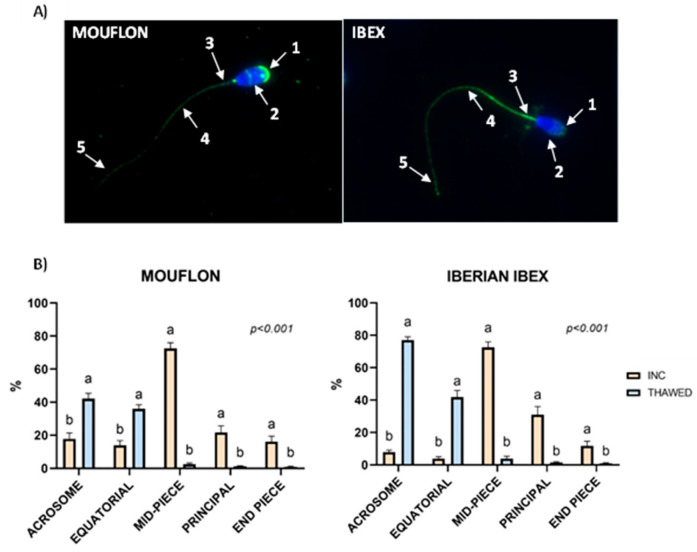
Immunolocalization of AQP3 in different membrane sperm domains. (**A**) Mouflon and Ibex spermatozoa with the presence of AQP3 in (1) the acrosome, (2) equatorial, (3) mid-piece, (4) principal, and (5) end piece. (**B**) Comparison of the means ± statistical error of AQP3 localization in mouflon and ibex after incubation (INC) with different treatments and after freezing-thawing (THAWED). The x-axis shows the region of the spermatozoa where AQP3 is located and the y-axis shows the percentage of AQP3. Different letters (a,b) indicate significant differences (*p* < 0.001).

No changes were found among treatments in the proportion of spermatozoa showing AQP3 in the different membrane domains after one hour of incubation and after freezing-thawing in both mouflon and ibex (Table 5, Table 6, Table 7 and Table 8). A relocation of AQP3 occurred in incubated samples after freezing. The expression of AQP3 in the acrosome and the equatorial region was greater (*p* < 0.001) in frozen-thawed samples than in incubated samples (Figure 3). Conversely, the expression of AQP3 in the mid-piece and principal piece strongly decreased (*p* < 0.001) after thawing (Figure 3).

## 3. Discussion

The present findings revealed, apparently for the first time, the presence of AR in the sperm membranes of wild and domestic small ruminants, adding weight to the notion that the receptor is not only present in the cytoplasm but also in the plasmalemma. Testosterone exerted a negative effect on sperm cryoresistance, enhancing the effects of the AQP blocker PHL, at least in mouflon, but did not affect AQP3 relocation.

We identified the presence of AR as a single band of about 48 KDa in all small ruminants studied. AR has been located at 85–87 KDa and 110 KD in human sperm [6,7], corresponding to isoforms AR-A and AR-B, respectively. A single band of approximately 110 KDa was observed in bank vole (*Clethrionomys glareolus*) sperm [9] and swine (*Sus scrofa domestica*) sperm [8]. However, in our study, the antibody used seems to detect the AR-NH2 domain of AR with a molecular weight of approximately 48 kDa [23]. Immunoblot analysis of cell lysates containing human AR, using the antibody named AR52, showed three predominant bands at 114, 88, and 71 kDa and a less prominent band at 48 kDa [24], the latter coinciding with the band detected in our study.

Our results along with previous reports show species-dependent variation in terms of AR location in spermatozoa. AR was identified in the proximal region of the head in bank vole spermatozoa [9]. In the present study, AR was located exclusively in the equatorial segment of the sperm head in all studied species. The localization of AR in the equatorial segment of spermatozoa may be related to its involvement in the fertilization process because the equatorial segment has a key role in the fusion of sperm with oocytes [25]. In the human and swine sperm, the AR is found at the midpiece [6,8], which is packed with mitochondria performing oxidative phosphorylation, and suggests that AR may be involved in sperm bioenergetic metabolism and motility in these species. Moreover, in bonnet monkeys (*Macaca radiate*), spermatozoa motility appears to be directly related to the distribution of testosterone-binding sites throughout the sperm surface [10].

The relative abundance of AR was greater at the onset of the rutting season, coinciding with high testosterone levels in mouflon [26], ibex [27], and goat [28]. These findings suggest that an increase in testosterone levels in peripheral blood leads to an increase in AR in the spermatozoa of these seasonal species. Conversely, in Merino ram, the greater expression was found at the end of the rutting season which could be explained, at least in part, by the fact that this breed presents a less-marked reproductive seasonality at temperate latitudes [13]. This season-dependent increase in AR has also been described in the Sertoli and Leydig cells of the testicle in domestic goose (*Anser anser domesticus*) [29], and roe deer (*Capreolus capreolus*) [30]. In summary, our findings support the fact that AR receptor is not only present in the cytoplasm but also in the plasmalemma, showing a seasonal expression in small ruminant species.

The results showed that mouflon sperm incubation with PHL along with testosterone (PHT+T group) produced the least favorable outcomes for most kinematic sperm variables in comparison with the Control group. We have demonstrated that, in mouflon sperm, short exposure to androgens produces a rapid effect. The fact that testosterone enhances the negative effect of the AQP inhibitor PHL in mouflon might be explained by the existence of a similar mechanism of action. Human sperm express a functional AR that can modulate the phosphoinositide-3 kinase (PI3K)/AKT pathway depending on the concentration of androgens, i.e., high androgen levels decrease PI3K activity [7]. Consequently, the reduction of PI3K activity interferes the sperm functionality (e.g., kinematic activity) [31] and survival (e.g., by activation of caspases) [7]. This process seems to underlie the rapid responses of sperm cells to androgens by a non-genomic mechanism. The inhibitory mechanism of the AQP blocker PHL is not well understood but it is likely a result of electrostatic interactions between the ligand and amino acid residues within the water channel [32], and reduction of water influx [33]. In addition, phloretin inhibits PI3K/AKT pathway [34], as previously reported for androgens [7]. In ibexes, this synergic effect of PHL and testosterone was not seen, probably by species-specific differences in the sensitivity to PHL concentrations used in our study.

A study by Martínez-Fresneda and coworkers has shown that the deleterious effect of freezing-thawing in acrosome is enhanced in the presence of high levels of testosterone in small ruminants [35]. In mouflons, the cryoresistance ratio for acrosome integrity was lower in the PHL+T than in Control and T groups. Once again, testosterone enhanced the negative effect of the AQP inhibitor PHL in this species. In ibex, the cryoresistance ratio for acrosome integrity was lower PHL+T, PHL, and T groups than in the Control group. Data suggest that blocking AQP3 with PHL affects the cryoresistance ratio for acrosome in a similar way that testosterone does (i.e., PI3K/AKT pathway) [34]. The location of AR in the equatorial segment of the sperm head appears to support this assumption. The question is how androgens can affect sperm cryoresistance through AQP3. AQP3 appears to be involved in the modulation of PI3K/AKT in breast cancer cells [36], and a similar condition in sperm cells should not be ruled out. Overall, the results suggest that AR exert a putative modulation pathway for cryoresistance through AQP3. The low number of animals used in this experiment was a limitation, affecting the power of the statistical test, but the model used in the present study, i.e., wild ruminants, does not allow the use of a large number of animals. However, the overall data provided valuable information about the role of testosterone concentrations on sperm response to freezing- thawing process.

A greater capacity of AQP3 relocation in mid- and principal pieces of the spermatozoa is linked to an increase in the osmo-adaptative capacity of ram ejaculates with a better capacity to withstand freeze-thawing processes [37]. Our data did not show differences in the location of AQP3 among the different experimental groups, but a relocation of AQP3 in the acrosome an equatorial region of the spermatozoa after freezing was seen. This suggests that androgens could affect the sperm cryoresistance in other different ways. The changes in the redistribution of AQP3 among incubated samples and incubated plus freeze-thawing ones do not coincide with the studies conducted with bulls [38], boar [39] and ram [17]. This could be due to variation among the different species under study and to the incubation process before freezing. Temperature [40], hypoxia [41], and ROS production [42] produce alterations in membrane fluidity and oxidative stress, that could predispose to AQP3 redistribution.

The cryoresistance index for the kinematic values of VCL, VSL, VAP, ALH, and BCF in ibex sperm treated with PDO plus testosterone was lower than in controls. PDO inhibits orthodox aquaporins (AQP1, AQP2, AQP4, and AQP5), and thus the results suggest that androgens might also exert their effects in cryoresistance through these orthodox aquaporins, modifying the transport of water, in addition to aquaglyceroporins as AQP3.

## 4. Materials and Methods

### 4.1. Animals

The animals studied were five Merino ram (*Ovis aries*) (7 years of age) and five Murciano-Granadina buck (*Capra hircus*) (7 years of age) and their respective wild ancestors, five Iberian ibexes (*Capra pyrenaica*) (5–7 years of age) and five mouflons (*Ovis gmelini*) (5–7 years of age) kept in captivity with natural photoperiod at the National Institute for Agricultural and Food Research and Technology (40°25′ N latitude; INIA-CSIC, Madrid, Spain). All handling procedures were approved by the INIA Ethics Committee (reference regional government PROEX 046.0/21) and performed following the Spanish Policy for Animal Protection RD53/2013, which conforms to European Union Directive 2010/63 regarding the protection of animals used in scientific experiments.

### 4.2. Semen Collection

In the domestic ruminants, semen collection was performed using an artificial vagina. In the wild ruminants, semen was collected by transrectal ultrasound-guided massage of the accessory glands (TUMASG) [43]. The wild ruminants were intravenously anesthetized using 50 μg/kg intravenous detomidine (Domosedan^®^) (Pfizer Inc., Amboise Cedex, France), 0.5 mg/kg ketamine hydrochloride (Imalgene-1000^®^) (Rhône Mérieux, Lyon, France) and 0.5 mg/kg tiletamine-zolazepam (Zoletil-100^®^) (Virbac España S.A., Barcelona, Spain). Anesthesia was maintained with 1.5% isoflurane (Isobavet^®^) (Intervet/Schering Plough Animal Health, Madrid, Spain) in oxygen (flow rate 2.5 L/min) administered via an endotracheal tube. Vital signs were monitored throughout the entire procedure. Real-time transrectal ultrasonography was used to examine the bulbourethral glands, seminal vesicles, and the ampulla of the vas deferens employing a 7.5 MHz linear array probe (Prosound 2, Aloka CO., LTD, Tokyo, Japan). TUMASG involved positioning the ultrasonographic probe on the ampulla of the vas deferens, employing a back-and-forth motion to induce sperm emission. In cases where the animal did not ejaculate, electrical stimuli (0.2 mA lasting 6–8 s) were administered using an electroejaculator, with intermittent breaks for TUMASG; typically, 1–3 electrical stimuli were necessary. The electroejaculator used was a Lane Pulsator IIIZ model (Lane Manufacturing Inc., Denver, CO, USA) consisting of a rectal probe 2.5 cm in diameter and 20.5 cm in length.

### 4.3. Experimental Procedure

Experiment 1. Identification and localization of AR

One ejaculate from each animal was collected at the onset of the rutting season (July for domestic ruminants and November for wild ruminants), coinciding with the peak plasma testosterone concentration, and at the end of the rutting season (February), when plasma testosterone concentrations were at their lowest for each species [26,27]. Western blotting (WB) and immunocytofluorescence (ICF) techniques were employed to identify the presence, distribution and quantification of AR in fresh spermatozoa.

WB: Samples for protein extraction were processed as described previously [17]. Briefly, after sperm centrifugation, the pellet was mechanically disrupted and incubated with lysis buffer made up of 1 mM benzamide, 125 mM Tris, 1% protease inhibitor cocktail, 6% sodium dodecyl sulfate (SDS), and 1 mM phenylmethylsulfonyl fluoride. Samples were centrifuged again at 8200 g for 5 min, and Laemmli-sample buffer (Tris, SDS, DTT, glycerol, b-mercaptoethanol, and bromophenol blue) was added to supernatant. Samples were boiled at 94 °C for 4 min, and aliquots of 35 μL were subsequently loaded onto 12% SDS-PAGE gels. Electrophoresis was run at 200 V for 50 min. Proteins from gels were subsequently transferred onto Amersham™ Protran^®^ 0.45 μm nitrocellulose membranes (Global Life Sciences Solutions, Buckinghamshire, UK) at 300 mA for 90 min. Then membranes were blocked for 1 h at room temperature with 5% BSA (Merck KGaA, Darmstadt, Germany) in PBS-Tween and incubated overnight at 4 °C with a dilution 1/2000 of the primary antibodies (AR-bs-0118R from Bioss Antibodies (Woburn, MA, USA)). The membranes were then washed three times in PBS-Tween and incubated with a secondary antibody (mouse anti-rabbit IgG-HRP, sc-2357) (Santa Cruz Biotechnology Inc., Dallas, TX, USA) at a dilution of 1/5000 at room temperature for 120 min, followed by extensive washing in PBS-Tween. Bands were visualized with WesternSure^®^ PREMIUM, LI-COR^®^ chemiluminescent substrate (Lincoln, NE, USA), using the C-DIGIT instrument (LI-COR Bio-sciences) and analyzed by the IMAGE STUDIO 4.0 software (LI-COR Bio-sciences). A Western blot of mouse vas deferens tissue lysate was performed to evaluate the specificity of the antibodies. A negative control WB was performed with lysed tissue from kidney, lung, heart, and liver, tissues with documented lack of AR (Figure S1) [44]. A total of three replicates per semen sample was made.

ICF: Spermatozoa were fixed with 4% paraformaldehyde, centrifuged (1200 g, 6 min), and the pellet resuspended in PBS to prepare smears on slides. The smears were allowed to dry, washed with PBS Tween, and covered (blocking) with 5% BSA in PBS for 60 min. The slides were washed and incubated with the primary antibody against AR (AR-bs-0118R from Bioss Antibodies (USA)), diluted 1/50 in PBS containing 0.1% Tween 20 and 1% BSA, at 4 °C overnight, in a wet chamber in the dark. After incubation, the smears were washed and incubated with the secondary antibody (polyclonal goat anti-rabbit Alexa Fluor 488 ab150077) (Abcam BV, Amsterdam, The Netherlands) diluted 1/500 in PBS containing 0.1% Tween 20 and 1% BSA, for 180 min in the dark at room temperature. Next, the slides were counterstained with 2.5 μg/mL of nuclear stain Hoechst 33342 (Sigma) for 5 min at room temperature. To check the specificity of the primary antibody we used the corresponding AR-blocking peptide. The samples were incubated with the AR primary antibody together with an AR-specific blocking peptide that was 20 times in excess. Finally, the slides were photographed by optical sectioning in fluorescence imaging (Zeiss Apotome 3) using an inverted Zeiss Axio Observer microscope at ×630 magnification, connected to a camera Zeiss Axiocam Mono (Oberkochen, Germany).

Experiment 2. In vitro assay of testosterone effect on cryoresistance and expression of AQP3

The putative effect of testosterone on sperm cryoresistance through AQP3 relocation was investigated in vitro in sperm samples with or without testosterone and subjected to different AQP3 inhibitors. This experiment was performed using ejaculates from the wild ancestors because they show a strong seasonal rutting season and the negative influence of high testosterone levels at the onset of the rutting season on cryoresistance is more evident. A total of 10 semen samples (5 from ibexes and 5 from mouflons) were used.

Sperm incubation: The following semen parameters were analyzed in fresh semen (0 h), after one hour of incubation, and after freezing-thawing. Sperm concentration was calculated using the Neubauer counting chamber (Marienfeld, Lauda-Königshofen, Germany). Sperm motility was evaluated using phase-contrast microscopy (Nikon (Eclipse 50i, Nikon Corporation, Tokyo, Japan) equipped with a camera (A312fc; Basler AG, Ahrensburg, Germany). Kinetic parameters of motility using computer-assisted sperm analysis (CASA) system and Sperm Class Analyzer v.4.0 software (Microptic S.L., Barcelona, Spain). Acrosome integrity (NAR) was assessed by fixing semen samples in 2% glutaraldehyde and observing 200 spermatozoa with a phase-contrast microscope at x1000 magnification. Sperm viability was measured using fluorescent probes with the Seminal Quality System SQS2 (Arquimea-Agrotech, Collado Villalba, Madrid, Spain). Lastly, the cryoresistance index ((Thawed value/Incubation value) × 100) was calculated for the different treatments studied for viability and kinetic sperm variables.

Once the seminal quality parameters of fresh semen were analyzed, seminal plasma was removed using the following washing solutions (TTG): Tes 210.6 mM, Tris 95.8 mM, glucose 10.1 mM) for mouflons, and TCG (Tris 313.7 mM, citric acid 104.7 mM, glucose 30.3 mM) for ibexes. The washed sperm samples were diluted 1:9 (*vol./vol.*) with the washing solution at 37 °C and centrifuged at 900 g for 20 min. After centrifugation, the supernatant was removed and the spermatozoa resuspended at room temperature in a Tyrode medium (NaCl 120 mM, KCl 1 mM, CaCl_2_ 2mM, MgSO_4_ 0.4 mM, Hepes 16.6 mM, sodium lactate 21.7 mM, sodium pyruvate 0.11 g/L, glucose 5.5 mM, and BSA 5 g/L; pH 7.5; 290 mOSm/kg) [35] at a final concentration of 25 million sperm/mL for mouflons and 100 million sperm/mL for ibexes.

To inhibit the AQPs, two kinds of inhibitors were used: 3-propanediol (PDO) which inhibits orthodox aquaporins (AQP1, AQP2, AQP4, and AQP5) by remaining within their pores due to its narrow diameter, and phloretin (PHL) which inhibits aquaglyceroporins (AQP3 and AQP7) by crossing the sperm plasma membrane, thanks to its hydrophobic nature, and binding to an internal binding site [45].

The samples were split into five aliquots and incubated for one hour at 38.5 °C in a humid atmosphere with 5% CO_2_. Five treatments were performed during the incubation. Tyrode with 10 µL of testosterone (T group) at a concentration of 6 ng/mL (Testosterone VETRANAL 46923, lot SZBA235XV; Sigma-Aldrich, Seelze, Germany), Tyrode with 10 µL of PDO (PDO group) at a concentration of 10 mmol/L (Sigma-Aldrich, St. Louis, MO, USA), Tyrode with 10 µL de PHL (PHL group) at a concentration of 500 µL/L (Sigma-Aldrich, St. Louis, MO, USA), Tyrode with 10 µL of PDO at a concentration of 10 mmol/L and 10 µL of testosterone (PDO+T group) at a concentration of 6 ng/mL and Tyrode with 10 µL of PHL (PHL+T group) at a concentration of 500 µL/L and 10 µL of testosterone at a concentration of 6 ng/mL. For the control, an aliquot solely extended in Tyrode’s solution was used.

Sperm cryopreservation: Sperm was frozen after incubation following the procedures described previously [46]. Mouflon sperm was extended with TTG containing 6% (*vol.*/*vol.*) egg yolk, while ibex sperm was extended with TCG containing 6% (*vol.*/*vol.*) egg yolk, both without any cryoprotectant. Extended samples were kept refrigerated for 1 h at 5 °C. Afterwards, the freezing diluent was added: TTG with 6% (*vol.*/*vol.*) egg yolk and 10% (*vol.*/*vol.*) glycerol for mouflons, or TCG with 6% (*vol.*/*vol.*) egg yolk and 10% (*vol.*/*vol.*) glycerol for Iberian ibex, with a final glycerol concentration of 5%. The suspension was allowed to equilibrate for 15 min at 5 °C. Past equilibration time, sperm samples were loaded into 0.25 mL straws (Minitüb GmbH, Haupstrasse 41, 84184, Tiefenbach, Germany), that were placed on a metal rack inside a polystyrene box to freezing in nitrogen vapors for 10 min at a height of 5 cm from it (freezing ramp: from 5 to −35 °C at 48 °C/min, from −35 to −65 °C at a rate of 22 °C/min, and from −65 to −93 °C at a rate of 3.5 °C). Finally, straws were submerged in liquid nitrogen for preservation. The straws were thawed one month later by placing them in a water bath at a temperature of 37 °C for 30 s.

IFC: AQP3 location in the membrane domains of spermatozoa [37] was analyzed. Briefly, the sperm samples were fixed in 4% paraformaldehyde. Cell suspensions were then centrifuged (1200 g, 6 min), and the pellet resuspended in PBS. The prepared smears were allowed to dry, washed with PBS-Tween, and covered (blocking) with 5% BSA in PBS for 60 min. The slides were washed and incubated with the primary antibody against AQP3 (ab125219 from Abcam, The Netherland), diluted 1/100 in PBS containing 0.1% Tween 20 and 1% BSA, at 4 °C overnight, in a wet chamber in the dark. After incubation, the smears were washed and incubated with the secondary antibody (polyclonal goat anti-rabbit Alexa Fluor 488) (Abcam BV, Amsterdam, The Netherlands) diluted 1/500 in PBS containing 0.1% Tween 20 and 1% BSA, for 180 min in the dark at room temperature. Next, the slides were counterstained with 2.5 μg/mL Hoechst 33342 (Sigma) for 5 min at room temperature. To check the specificity of the primary the samples were incubated with the AQP3 primary antibody together with an AQP3-specific blocking peptide that was five times in excess, as well as omission of the AQP3 primary antibody. The slides were photographed by optical sectioning in fluorescence imaging (Zeiss Apotome 3) using an inverted Zeiss Axio Observer microscope at ×630 magnification, connected to a camera Zeiss Axiocam Mono. Furthermore, the proportion of spermatozoa showing AQP3 in different plasma membrane domains was assessed by examining 200 cells per sample, using a Nikon Eclipse E200 epifluorescence light microscope (Nikon Instruments Inc., New York, NY, USA).

### 4.4. Statistical Analysis

The values of the relative abundance of AR, in sperm cells and the percentage of AQP3 with a skewed distribution (determined by the Shapiro-Wilk test) were transformed before statistical analysis. Values of AR abundance and the cryoresistance index were log-transformed and the percentage of AQP3 and sperm variables, before and after incubation and freeze-thawing, were arcsine-transformed. The influence of season on the relative abundances of AR (experiment 1) for ram, buck, mouflon and ibex was analyzed by one way-ANOVA following the statistical model xij = m + Ai + eij, where xij = relative abundances of AR, m = the overall mean of variable x, Ai = the effect of season (i = 1–2 onset and end of the rutting season) and eij = the residual (l–5). When data were not normally distributed despite log transformation, a non-parametric test (Mann-Whitney U Test) was used. The influence of treatment with testosterone and AQPs blockers on sperm variables (experiment 2) was analyzed by one way-ANOVA following the statistical model xijk = m + Ai + Bj + ABij + eijk, where xijk = the sperm variable, m = the overall mean of variable x, Ai = the effect of treatment (i = 1–6 treatments: controls, T, PDP, PDO+T, PHL, PHL+T), eijk = the residual (1–5). A post hoc Fisher test was performed to compare differences between groups. Differences in the proportion of sperm with AQP3 location in the different membrane domains between incubated and frozen-thawed samples were also compared by ANOVA. Where applicable, significance was set at *p* ≤ 0.05. All statistical calculations were undertaken using Statistica software for Windows (v. 9.1, series 2010, StatSoft Inc., Tulsa, OK, USA).

## 5. Conclusions

In conclusion, we have identified the presence of AR in the equatorial domain of the plasma membrane of wild and domestic small ruminants. The negative effect of testosterone on sperm cryoresistance is not carried out through AQP3 relocation.

## Figures and Tables

**Table 1:** Kinetic parameters and acrosome integrity (NAR) (mean ± SEM) of mouflon at time 0 h (fresh) and after 1 h of incubation (Inc. 1 h) with different treatments: Control, testosterone (T), the inhibitors 1,3-propanediol (PDO) and phloretin (PHL), and the inhibitors with testosterone (PDO+T and PHL+T).

	FRESH	Inc. 1 h CONTROL	Inc. 1 h T	Inc. 1 h PDO	Inc. 1 h PDO+T	Inc. 1 h PHL	Inc. 1 h PHL+T
Viability (%)	55.4 ± 6.7	52.4 ± 6.4	48.6 ± 9.3	45 ± 10.9	42 ± 14	43.6 ± 11	38.2 ± 13.8
NAR (%)	92 ± 2.1 ^a^	71.4 ± 5.9 ^ab^	63.6 ± 10 ^b^	66 ± 9.4 ^b^	59 ± 12.5 ^b^	63.6 ± 8.1 ^b^	57.6 ± 13.3 ^b^
Motile progressive (%)	50.7 ± 7.6	30.4 ± 11.6	45.6 ± 15.1	36 ± 10.6	45.9 ± 14.4	26.3 ± 9.6	23.3 ± 14.5
Total motile (%)	63.4 ± 7.9	40.7 ± 13.5	56.7 ± 14.2	51.1 ± 12.5	53.6 ± 14.9	39.6 ± 9.4	38.6 ± 14.2
VCL (µm/s)	141 ± 19.9 ^a^	97 ± 19 ^ab^	105.8 ± 19.2 ^ab^	103.2 ± 25.7 ^ab^	117.2 ± 29.1 ^ab^	103.7 ± 23.8 ^ab^	75.2 ± 32.8 ^b^
VSL (µm/s)	75.2 ± 11 ^a^	60.3 ± 11.6 ^a^	68.9 ± 14.1 ^a^	70.3 ± 19.5 ^a^	84.4 ± 25 ^a^	39.8 ± 10.5 ^ab^	31 ± 20 ^b^
VAP (µm/s)	92.7 ± 12.5 ^a^	71.2 ± 13.9 ^a^	81.8 ± 16.2 ^a^	81.7 ± 21.4 ^a^	97.7 ± 26.4 ^a^	62.7 ± 13.7 ^ab^	45.1 ± 25 ^b^
LIN (%)	54.2 ± 3.3 ^a^	58.5 ± 3.3 ^a^	59.5 ± 7.3 ^a^	58.3 ± 9.5 ^a^	57.9 ± 13.7 ^a^	38.1 ± 8.8 ^ab^	24.3 ± 11.7 ^b^
STR (%)	77.8 ± 2.3 ^a^	78 ± 2.3 ^a^	74.4 ± 7.4 ^a^	73.3 ± 8.5 ^a^	68.9 ± 15.7 ^a^	56.7 ± 10.2 ^ab^	39.5 ± 14 ^b^
WOB (%)	68 ± 3.1 ^a^	70.6 ± 3.8 ^a^	73.5 ± 6.1 ^a^	72.2 ± 8.3 ^a^	76.9 ± 5.9 ^a^	60.6 ± 5.3 ^ab^	46.6 ± 10.8 ^b^
ALH (µm)	2.7 ± 0.4	1.9 ± 0.3	1.8 ± 0.2	1.7 ± 0.3	1.8 ± 0.2	2.5 ± 0.6	1.8 ± 0.5
BCF (Hz)	22 ± 3.2 ^a^	18.4 ± 2.2 ^a^	18.3 ± 3.3 ^a^	16.7 ± 4.3 ^ab^	18.1 ± 4.4 ^ab^	13 ± 2.7 ^ab^	9.5 ± 5 ^b^

Different lowercase letters (a, b) indicate significant differences (*p* < 0.05) between treatments for each parameter studied. (VCL: curvilinear velocity, VSL: straight-line velocity, VAP: average path velocity, LIN: linearity index, STR: straightness index, WOB: wobble index, ALH: amplitude of lateral head displacement, and BCF: beat-cross frequency).

**Table 2:** Kinetic parameters and acrosome integrity (NAR) (mean ± SEM) of Iberian ibex at time 0 h (fresh) and after 1 h of incubation (Inc. 1 h) with different treatments: control, testosterone (T), the inhibitors 1,3-propanediol (PDO), and phloretin (PHL), and the inhibitors with testosterone (PDO+T and PHL+T).

	FRESH	Inc. 1 h CONTROL	Inc. 1 h T	Inc. 1 h PDO	Inc. 1 h PDO+T	Inc. 1 h PHL	Inc. 1 h PHL+T
Viability (%)	69.4 ± 9.4	65.5 ± 4.5	65.3 ± 6.3	65.8 ± 6.9	65.8 ± 5.1	58 ± 6.9	52.3 ± 5.5
NAR (%)	94.8 ± 2.1 ^a^	83.5 ± 6 ^b^	82.3 ± 5.4 ^b^	85 ± 3 ^ab^	84.5 ± 4.4 ^ab^	74.8 ± 6.5 ^b^	76.3 ± 6.4 ^b^
Motile progressive (%)	32.2 ± 11.6 ^a^	24.5 ± 10.6 ^abc^	23.7 ± 11.2 ^abc^	29.1 ± 17.1 ^abc^	34.8 ± 17.4 ^ac^	2.1 ± 1.2 ^b^	3.6 ± 2.7 ^bc^
Total motile (%)	57.8 ± 8.4 ^a^	49 ± 9.9 ^ab^	47.5 ± 10.6 ^ab^	51.4 ± 12.8 ^ab^	52 ± 17.9 ^ab^	19.8 ± 8.4 ^b^	27.7 ± 6.9 ^ab^
VCL (µm/s)	100 ± 17.2 ^a^	71.7 ± 16.7 ^a^	72.1 ± 18.8 ^a^	86.3 ± 26 ^a^	96.7 ± 20.7 ^a^	27.8 ± 6.4 ^b^	32.2 ± 4.2 ^b^
VSL (µm/s)	32.2 ± 9.8 ^a^	27.5 ± 12.6 ^a^	32.8 ± 16.3 ^a^	39.9 ± 21.8 ^a^	39.6 ± 18 ^a^	4.2 ± 1.2 ^b^	4 ± 0.6 ^b^
VAP (µm/s)	55.1 ± 13 ^a^	41.8 ± 14.9 ^a^	45.8 ± 18.4 ^a^	55.6 ± 25.7 ^a^	59.1 ± 21.3 ^a^	11.2 ± 3.1 ^b^	13.5 ± 2.4 ^b^
LIN (%)	29.4 ± 4.7 ^ab^	29.4 ± 6.2 ^ab^	32.8 ± 7.8 ^a^	34.6 ± 10.2 ^a^	32.6 ± 9.6 ^a^	14.9 ± 1.4 ^ab^	12.2 ± 1.1 ^b^
STR (%)	50.5 ± 4.7	49.9 ± 6.5	53.5 ± 6.6	55.7 ± 8.5	54.4 ± 9.9	37 ± 2.5	29.5 ± 3.2
WOB (%)	54.8 ± 4.3 ^ab^	52.7 ± 4.8 ^ab^	55 ± 5.9 ^a^	56.7 ± 8.4 ^a^	56.4 ± 7.1 ^ab^	38.8 ± 1.2 ^b^	41.8 ± 2.1 ^ab^
ALH (µm)	2.4 ± 0.3 ^a^	1.8 ± 0.2 ^ab^	1.7 ± 0.2 ^b^	2 ± 0.2 ^ab^	2.3 ± 0.1 ^ab^	1.1 ± 0.2 ^c^	1.2 ± 0.1 ^c^
BCF (Hz)	12 ± 2.6 ^a^	9.4 ± 2.6 ^a^	10.7 ± 2.9 ^a^	12.6 ± 4.1 ^a^	13.6 ± 3.9 ^a^	2.1 ± 0.8 ^b^	2.8 ± 0.7 ^b^

Different lowercase letters (a, b, c) indicate significant differences (*p* < 0.05). (VCL: curvilinear velocity, VSL: straight-line velocity, VAP: average path velocity, LIN: linearity index, STR: straightness index, WOB: wobble index, ALH: amplitude of lateral head displacement, and BCF: beat-cross frequency).

**Table 3:** Cryoresistance index (ICR) (Mean ± SEM) of sperm parameters of mouflon with different treatments: control, testosterone (T), inhibitors 1,3-propanediol (PDO) and phloretin (PHL), and inhibitors with testosterone (PDO+T and PHL+T).

	ICR CONTROL	ICR T	ICR PDO	ICR PDO+T	ICR PHL	ICR PHL+T
Viability (%)	11.3 ± 5.1	10.2 ± 6.3	12.3 ± 5.3	13.7 ± 9.2	6.9 ± 3.1	15.6 ± 6.3
NAR (%)	27.1 ± 6 ^a^	25.9 ± 3.5 ^a^	22.3 ± 7.2 ^ab^	19.3 ± 3.5 ^ab^	19.6 ± 2 ^ab^	11.7 ± 4.4 ^b^
Motile progresive (%)	15.6 ± 5.6	16.9 ± 11.4	24.2 ± 19	4.8 ± 1.8	9.3 ± 3.8	6.5 ± 5
Total motile (%)	20.3 ± 4.9	14.7 ± 7.8	20.7 ± 14.3	23.1 ± 15.9	12.9 ± 4	28.6 ± 9.3
VCL (µm/s)	83.8 ± 5.3	77.3 ± 14.5	64.7 ± 18.3	81.1 ± 14.4	86.2 ± 6	89.5 ± 7.5
VSL (µm/s)	39.6 ± 3.7 ^ab^	53.8 ± 15.2 ^ab^	44.2 ± 17.6 ^b^	51.7 ± 15.1 ^ab^	84.3 ± 9.7 ^a^	80.6 ± 11.9 ^a^
VAP (µm/s)	57.6 ± 2.5	60.1 ± 14	53.1 ± 17.4	62.3 ± 14.4	80.8 ± 9.2	83.4 ± 10.2
LIN (%)	48.2 ± 5.2 ^b^	64.8 ± 11.5 ^ab^	52.6 ± 17.2 ^b^	57.2 ± 12.1 ^ab^	90 ± 6.4 ^a^	88.1 ± 9.5 ^a^
STR (%)	75 ± 4.7 ^ab^	79.6 ± 11.6 ^ab^	62.2 ± 17.6 ^b^	76.1 ± 9 ^ab^	97.8 ± 2.2 ^a^	93.4 ± 4.9 ^a^
WOB (%)	69.6 ± 5 ^ab^	78.2 ± 5.8 ^ab^	64.4 ± 17.3 ^b^	70.3 ± 8.3 ^ab^	88 ± 5.3 ^ab^	91.5 ± 6 ^a^
ALH (µm)	99.3 ± 0.7	91.5 ± 8.5	78.1 ± 19.6	91.8 ± 8.2	91 ± 5.5	95.7 ± 4.3
BCF (Hz)	56.9 ± 6	64.4 ± 16.5	57.3 ± 17.4	72.6 ± 13.3	87.5 ± 7.6	84.9 ± 9.2

Lowercase letters (a, b) indicate significant differences (*p* < 0.05) between treatments for each of the studied parameters, with the letter “a” indicating the highest value. (VCL: curvilinear velocity, VSL: straight-line velocity, VAP: average path velocity, LIN: linearity index, STR: straightness index, WOB: wobble index, ALH: mean amplitude, and BCF: beat-cross frequency).

**Table 4:** Cryoresistance index (ICR) (Mean ± SEM) of sperm parameters of Iberian ibex males with different treatments: control, testosterone (T), inhibitors 1,3-propanediol (PDO) and phloretin (PHL), and inhibitors with testosterone (PDO+T and PHL+T).

	ICR CONTROL	ICR T	ICR PDO	ICR PDO+T	ICR PHL	ICR PHL+T
Viability (%)	27.5 ± 6.5	31.7 ± 5.8	20.8 ± 4.8	18.6 ± 9.1	17.8 ± 10.8	39.7 ± 6.5
NAR (%)	45.2 ± 2.2 ^a^	33.8 ± 4.1 ^b^	40.4 ± 4.8 ^ab^	26.5 ± 9 ^ab^	16.8 ± 9.9 ^b^	28 ± 4.5 ^b^
Motile progressive (%)	10.6 ± 3.8	24.6 ± 5.9	18.9 ± 2.9	5.3 ± 3.1	26.3 ± 20.8	38.1 ± 22.2
Total motile (%)	12.9 ± 2.6 ^b^	22.3 ± 4.1 ^b^	22 ± 2.3 ^b^	35.7 ± 21.4 ^ab^	59.6 ± 23.3 ^a^	29.3 ± 8.2 ^ab^
VCL (µm/s)	85.2 ± 7.1 ^a^	87.4 ± 7.1 ^a^	91.7 ± 8.1 ^a^	54.5 ± 10.1 ^b^	96.5 ± 3.5 ^a^	100 ± 0 ^a^
VSL (µm/s)	80.9 ± 9.4 ^a^	77.7 ± 11.2 ^a^	76.6 ± 15.2 ^a^	45 ± 9.5 ^b^	100 ± 0 ^a^	100 ± 0 ^a^
VAP (µm/s)	82.7 ± 7.9 ^a^	81.5 ± 9.5 ^a^	79.5 ± 8.4 ^a^	49.2 ± 5.4 ^b^	100 ± 0 ^a^	100 ± 0 ^a^
LIN (%)	96.2 ± 2.9 ^ab^	78.1 ± 7.2 ^b^	79.8 ± 11.8 ^ab^	78.7 ± 12.2 ^ab^	97 ± 3 ^ab^	100 ± 0 ^a^
STR (%)	100 ± 0	90.7 ± 4.2	88.2 ± 10.2	86.9 ± 10.3	95.8 ± 4.2	100 ± 0
WOB (%)	90.6 ± 2.4 ^ab^	87.1 ± 4.6 ^b^	87.8 ± 6.7 ^b^	86.8 ± 4.5 ^b^	100 ± 0 ^a^	100 ± 0 ^a^
ALH (µm)	88.7 ± 5 ^a^	95.7 ± 2.8 ^a^	94.3 ± 4.2 ^a^	69.9 ± 12.3 ^b^	95.7 ± 4.3 ^a^	100 ± 0 ^a^
BCF (Hz)	88.8 ± 5.2 ^a^	86.1 ± 7.8 ^a^	76.7 ± 12.8 ^a^	49.8 ± 18 ^b^	100 ± 0 ^a^	100 ± 0 ^a^

Lowercase letters (a, b) indicate significant differences (*p* < 0.05). (VCL: curvilinear velocity, VSL: straight-line velocity, VAP: average path velocity, LIN: linearity index, STR: straightness index, WOB: wobble index, ALH: mean amplitude, and BCF: beat-cross frequency).

**Table 5:** AQP3 location (Mean ± SEM) in mouflon spermatozoa at time 0h and after incubation (Inc. 1 h) with different treatments: control, testosterone (T), the inhibitors 1,3-propanediol (PDO) and phloretin (PHL), and the inhibitors with testosterone (PDO + T, and PHL + T).

	FRESH	Inc. 1 h CONTROL	Inc. 1 h T	Inc. 1 h PDO	Inc. 1 h PDO+T	Inc. 1 h PHL	Inc. 1 h PHL+T
Acrosome (%)	2.7 ± 1.6	14.9 ± 9.2	14.1 ± 8	16.3 ± 7.2	22.4 ± 11.4	16.1 ± 7.7	23.3 ± 11.2
Equatorial (%)	0.7 ± 0.5	11.9 ± 8.5	11.5 ± 7	14.7 ± 7.1	15.1 ± 7.8	13.2 ± 6.6	17.3 ± 8.1
Mid-piece (%)	68.1 ± 4.1	83.4 ± 7	68.5 ± 7.4	73.2 ± 13.5	64.4 ± 9.6	71.9 ± 6.7	73.3 ± 6.9
Principal (%)	22.2 ± 10.8	19.1 ± 9.8	19.6 ± 9.3	26.8 ± 12.6	19.6 ± 8.6	21.7 ± 10.4	23.4 ± 13.3
End piece (%)	12 ± 5.4	12.3 ± 6.8	15 ± 7.1	17.4 ± 9.2	14.1 ± 6.4	16.7 ± 9	21.1 ± 13

**Table 6:** Mean ± SEM of the localization of aquaporin 3 (AQP3) in Iberian ibex spermatozoa at time 0 h and after incubation (Inc. 1 h) with different treatments: control, testosterone (T), the inhibitors 1,3-propanediol (PDO), and phloretin (PHL), and the inhibitors with testosterone (PDO+T and PHL +T).

	FRESH	Inc. 1 h CONTROL	Inc. 1 h T	Inc. 1 h PDO	Inc. 1 h PDO + T	Inc. 1 h PHL	Inc. 1 h PHL+T
Acrosome (%)	2.4 ± 0.9 ^c^	8.9 ± 1.6 ^ab^	9.3 ± 3.9 ^ab^	3.5 ± 1.8 ^bc^	5.4 ± 1.6 ^abc^	12.9 ± 3.9 ^a^	6.1 ± 3.2 ^abc^
Equatorial (%)	0.4 ± 0.3	3 ± 1.4	5 ± 3.2	1.2 ± 1.2	1.2 ± 0.4	6.7 ± 3.8	4.7 ± 2.7
Mid-piece (%)	72.3 ± 6.9	63.6 ± 7	70.5 ± 9.8	87.7 ± 5.6	77.9 ± 6.4	70.5 ± 7.9	70.3 ± 11.5
Principal (%)	21.1 ± 8	29.6 ± 12.2	34.5 ± 13.4	35.5 ± 18.5	36 ± 15	31.4 ± 4.6	17.6 ± 12.1
End piece (%)	6.5 ± 2.7	12.7 ± 5.9	15.1 ± 9.1	15.2 ± 8.5	16.2 ± 8.5	4.4 ± 1.6	5.1 ± 3.6

Lowercase letters (a, b, and c) denote significant differences (*p* < 0.05) between treatments for each of the studied parameters.

**Table 7:** Mean ± SEM of aquaporin 3 (AQP3) localization in mouflon spermatozoa thawed with different treatments: control, testosterone (T), the inhibitors 1,3-propanediol (PDO) and phloretin (PHL), and the inhibitors with testosterone (PDO+T and PHL+T).

	THAWED CONTROL	THAWED T	THAWED PDO	THAWED PDO+T	THAWED PHL	THAWED PHL+T
Acrosome (%)	41.04 ± 10.4	42.51 ± 5.73	41.86 ± 10.46	37.74 ± 7.98	45.18 ± 8.47	45.11 ± 5.66
Equatorial (%)	32.46 ± 6.56	36.62 ± 4.32	41.04 ± 6.71	34.25 ± 7.95	35.35 ± 4.76	36.59 ± 7.14
Mid-piece (%)	3.84 ± 1.6	0.8 ± 0.58	3.86 ± 2.82	2.04 ± 1.35	0.99 ± 0.44	3.62 ± 2.42
Principal (%)	0.92 ± 0.83	0.8 ± 0.8	1.09 ± 0.71	1.65 ± 1.65	1 ± 1	1.2 ± 0.97
End piece (%)	0.92 ± 0.18	0.8 ± 0.58	1.07 ± 0.84	2.27 ± 2.27	0.4 ± 0.4	0.6 ± 0.6

**Table 8:** Mean ± SEM of aquaporin 3 (AQP3) localization in ibex spermatozoa thawed with different treatments: control, testosterone (T), the inhibitors 1,3-propanediol (PDO) and phloretin (PHL), and the inhibitors with testosterone (PDO+T and PHL+T).

	THAWED CONTROL	THAWED T	THAWED PDO	THAWED PDO+T	THAWED PHL	THAWED PHL+T
Acrosome (%)	78.4 ± 3.9	76.9 ± 4.3	80.2 ± 6.2	72.7 ± 8.8	75 ± 6	78.7 ± 4.7
Equatorial (%)	50.1 ± 11.4	43.6 ± 8.8	40.9 ± 14	30.9 ± 12.8	45 ± 9.4	38.5 ± 5.9
Mid-piece (%)	2.9 ± 1.8	4.8 ± 4	6.9 ± 6.9	1.2 ± 1.2	3.1 ± 2.8	4.6 ± 4.6
Principal (%)	1.7 ± 1.3	2 ± 1.3	0.8 ± 0.8	0.2 ± 0.2	1.4 ± 1.1	2.4 ± 2.4
End piece (%)	1.4 ± 0.9	1.2 ± 0.7	0.8 ± 0.8	0 ± 0	1.2 ± 1.2	1.2 ± 1.2

## Data Availability

The data that support the findings of this study are available on request from the corresponding author.

## Supplementary Materials

The following supporting information can be downloaded at: https://www.mdpi.com/article/10.3390/ijms252211972/s1.
