# Supplementary material for: Detection of Androgen Receptors in Spermatozoa of Small Ruminants: A Putative Modulation Pathway for Cryoresistance Through AQP3
Source: Int J Mol Sci. 2024 Nov 7;25(22):11972. doi: 10.3390/ijms252211972 (PMC11593671; doi:10.3390/ijms252211972)
Supplement: Supplementary file 1 [file ijms-25-11972-s001.zip › ijms-3254940-supplementary.pdf]

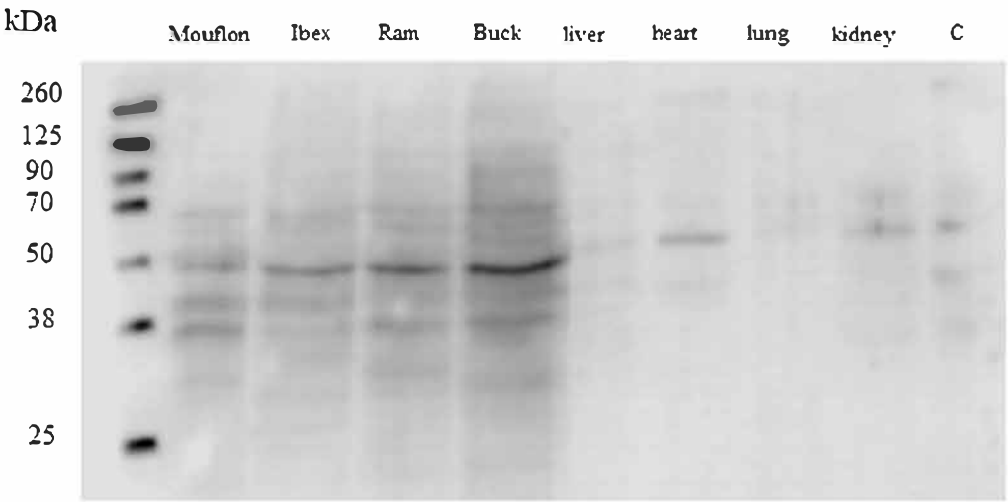

Figure S1 Negative control of AR. Representative Immunoblots for AR in mouflon, ibex, Merino ram and Murciano- Granadina buck spermatozoa. AR band is about 48 kDa. A negative control WB was performed with lysed tissue from liver, heart, lung, and kidney tissues with documented lack of AR.
